# A Comparative Study on the Physicochemical and Antioxidant Properties of Honeys From *Apis mellifera* L. and *Meliponula beccarii* L. Collected From Western Oromia, Ethiopia

**DOI:** 10.1155/2024/4448277

**Published:** 2024-10-21

**Authors:** Ofijan Tesfaye, Diriba Muleta, Asnake Desalegn

**Affiliations:** ^1^Oromia Agricultural Research Institute, Haro Sebu Agricultural Research Center, Haro Sebu, Oromia, Ethiopia; ^2^College of Natural and Computational Sciences, Institute of Biotechnology, Addis Ababa University, Addis Ababa, Ethiopia; ^3^Department of Microbial, Cellular and Molecular Biology, College of Natural and Computational Sciences, Addis Ababa University, Addis Ababa, Ethiopia

**Keywords:** *A. mellifera*, antioxidant, market honey, physicochemical composition, stingless bee

## Abstract

Honey is a natural substance synthesized by honeybees. Its physicochemical properties and antioxidant activities differ among honey types due to floral and entomological origins. This is a comparative study on the physicochemical and antioxidant properties of honey from *Apis mellifera* and *Meliponula beccarii* L. (stingless bee) collected from different sources. *A. mellifera* honey samples were collected from hives (*n* = 13) and local markets (*n* = 13). *M. beccarii* honey samples were collected from local markets (*n* = 13). The honey samples were designated as *A. mellifera* fresh honey directly collected from hives (AMFH), *A. mellifera* honey collected from the local markets (AMMH), and stingless bees (*M. beccarii*) honey collected from markets (MBH). Physicochemical and antioxidant properties were analyzed using standard protocols. The antioxidant properties of the honey samples were assessed using total phenolic, flavonoids, and 2,2-diphenyl-1-picrylhydrazyl (DPPH) methods. Honey from the stingless bee (MBH) had significantly (*p* < 0.0001) higher (33.5 ± 3.0%) moisture content, free acidity (113 ± 5.0 meq/kg), and maltose (3.1 ± 1.0), but significantly lower (*p* < 0.001) levels of hydroxymethyl furfural (4.4 ± 2.0 mg/kg), pH (3.0 ± 0.0), EC (0.25 ± 0.0 mS/cm), fructose (19.6 ± 2.4%), glucose (18.2 ± 1.62%), and sucrose (0.18 ± 0.13, *p* < 0.05) compared to *A. mellifera* honey collected from markets. Honey from the stingless bees had higher phenolic (273 ± 9.0 mgGAE/100 g), flavonoid (41 ± 21 mgQE/100 g), and antioxidant content (104 ± 6.0 mgAAE/100 g); however, the differences were not statistically significant (*p* > 0.05). Honey samples from the stingless bees had higher moisture, phenolic, flavonoid, and antioxidant contents but lower pH, HMF, sugar, ash, and electrical conductivity compared to *A. mellifera* honeys collected from markets. *A. mellifera* honey collected directly from the hive had higher quality than those purchased from markets. A strong awareness creation program is needed for consumers as well as honey producers to maintain the quality of honey.

## 1. Introduction

Bees are grouped under the family Apidae but are categorized under subfamilies *Apinae* and *Meliponaie*, respectively. Honeybees consist of 11 species, genus *Apis*, with *Apis mellifera* as the most extensive species in the world. There are more than 500 species of stingless bees, *Trigona* and *Meliponula* (in Africa), while *Melipona* (in the United States) are the two most extensive genera found in the world [1]. According to Pauly and Zewdu [2], in Ethiopia, *M. beccarii*, *Liotrigon abottegoi*, *Liotrigona baleensis*, *Hypotrigona gribodoi*, *Hypotrigona ruspolii*, and *Plebeina armata* are the stingless wild bees. *Trigona* species make their honey in tree trunks, while *M. beccarii* is in the underground nests [2].

Honey is a natural product made by honeybees from the nectar of different flowering plants. It is composed of sugar solution in combination with minerals, vitamins, enzymes, free amino acids, flavoring agents, and several volatile organic compounds [3, 4]. The physicochemical properties of a given honey are influenced by several factors, such as the type of nectar, bee species, geographical conditions (climatic and soil), and postharvest honey handling practices [5].

According to the Codex Alimentarius Commission [6] and ESA [7], foreign food ingredients shall not be added to honey, especially during processing and storage. To determine the quality of honey and detect possible adulterants, analysis of its physicochemical properties is essential [8, 9].

Honey is reported to contain antioxidant compounds capable of scavenging free radicals that could induce oxidative damage to cells and cellular components [10–12]. These antioxidant compounds in the honey are reported to be the plant secondary metabolites ingested by the honeybees during visits to the forage plants [13]. Honey has anticancer, anti-inflammatory, and antimicrobial properties and a role in protecting chronic diseases [14–16]. Comparative analysis of the physicochemical, proximate, and antioxidant characteristics of honey from stingless bees (*Meliponula beccarii* L.) from modern and wild hives was reported by Negera, Degu, and Tigu [17]. Tigistu, Worku, and Mohammed [18] also compared the physicochemical composition of *A. mellifera* L. honeys from farmers and shops in the southern part of the country. The therapeutic and antioxidant nature of stingless bees might be linked to the newly discovered disaccharide “trehalulose” [19–22].

The study areas from where the honey samples were collected have a high potential for the production of honey since they are endowed with a huge diversity of flora and high populations of *A. mellifera* as well as *M. beccarii.* Such conditions made the area one of the most prominent honey production sites all year round. To the best of our knowledge, there have been few studies conducted to assess the physicochemical properties and antioxidant activities of honey produced in the country. The only comparative studies the authors retrieved from published sources in the country for *A. mellifera* and *M. beccarii*, respectively, were those reported by Tigistu, Worku, and Mohammed [18] and Negera, Degu, and Tigu [17]. The comparative study done by the later authors was only on fresh honey from modern and natural hives and did not look into commercial honey from markets. Therefore, the aim of this study was to compare the physicochemical composition and antioxidant properties of fresh honey directly collected from hives and commercial honey from markets. The comparative study was made both for *A. mellifera* and *M. beccarii* from different sources.

## 2. Materials and Methods

### 2.1. Honey Sample Collection

**Figure 1:**
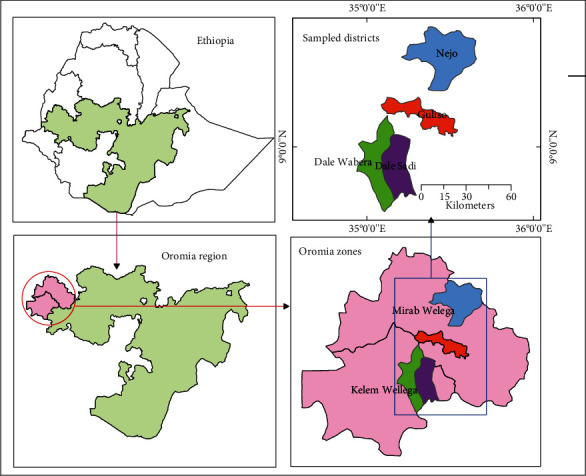
Map showing areas of honey sample collection.

The honey samples were collected from potential honey production areas of Kellem Wollega Zone (Hawetu gandaso, Hawetu birbir, and Kombo Kebeles of Haro Sabu district) and West Wollega Zone (Lalisa qami, Moga kobore, and Kurfessa birbir kebeles of Gulisso district). “Kebele” is a term in a local language that represents the smallest administrative region. For this work, three sources of honey samples, namely, (1) *A. mellifera* fresh honey directly collected from hives (AMFH), (2) *A. mellifera* honey collected from the local markets (AMMH), and (3) *M. beccarii* honey collected from markets (MBH), collected from markets (MBH) of the study areas, were analyzed (Figure 1). For each source, 13 honey samples and a total of 39 honey samples were collected. The collected honey samples were brought to the Holata Apiculture Research Center and Addis Ababa Center for Food Science and Nutrition using sterile containers.

### 2.2. Physicochemical Properties

#### 2.2.1. Moisture Content

The moisture content of honey samples was determined using an Abbérefractometer (ABBE-5 Bellingham Stanley. Ltd, United Kingdom) adjusted at 20°C and regularly calibrated with distilled water. Honey samples were homogenized and placed in a water bath until all the sugar crystals were dissolved. After homogenization, the surface of the prism of the refractometer was covered with honey, and after 2 min, the refractive index was determined. The value of the refractive index of the honey sample was determined using a standard table designed for this purpose [23].

#### 2.2.2. pH and Free Acidity

From each honey sample, 10 g was dissolved in 75 mL of distilled water in a 250 mL beaker and stirred using a magnetic stirrer. The electrode of the pH meter (Mettler Toledo, China) was immersed in the solution, and the pH of honey was recorded. For determination of the free acidity, the solution was titrated with 0.1 M sodium hydroxide (NaOH) solution to pH 8.30. For precision, the reading to the nearest 0.2 mL was recorded using a 10 mL Burette. Free acidity is expressed as mill equivalents or a millimole of acid per kilogram of honey and is equal to a millimeter of 0.1 M NaOH × 10. The result is expressed to one decimal place following the procedure of [23].

 $$\begin{array}{c} \text{Acidity}=10\;V \end{array}$$

where *V* is the volume of 0.1 N NaOH in 10 g of honey.

#### 2.2.3. Determination of Total Ash Content

Determination of ash content was carried out by incinerating honey samples at 600°C in a muffle furnace (BioBase JKKZ.5.12GJ, Shandong Ltd., China) to constant mass [23]. First, the ash dish was heated in an electrical muffle furnace at ashing temperature and subsequently cooled in a desiccator to room temperature and weighted to 0.001 g (M2). Then, 5 g (M0) of each honey sample was weighed to the nearest 0.001 g and taken into a platinum dish, and two drops of olive oil were added to prevent foaming. Water was removed and started ashing without loss at a low heat rising to 350–400°C using electrical devices. After the preliminary ashing, the dish was placed in the preheated furnace and heated for at least 1 h. The ash dish was cooled in the desiccators and weighed. The ashing procedure was continued until a constant weight was reached (M1). Lastly, the percentage of weight of ash in g/100 g honey was calculated using the following formula:

 $$\begin{array}{c} \text{WA}=\frac{M1-M2}{\text{M}0} \end{array}$$

where *M*0 is the weight of honey taken, *M*1 is the weight of ash+dish, and *M*2 is the weight of the dish.

#### 2.2.4. Determination of Sugars

Honey sugars were determined using high-performance liquid chromatography (HPLC-1260 Infinity Series Agilent Technologies, Germany). Five grams of honey was dissolved in 40 mL of water. Twenty-five milliliters of acetonitrile was pipetted into a 100-mL volumetric flask, and the honey solution was transferred to a flask and filled to the mark with distilled water. The solution of each honey sample was filtered using a syringe filter (0.45 *μ*m) before chromatographic analysis. The HPLC separation system was composed of an analytical stainless steel column, 4.6 mm in diameter and 250 mm in length, containing amine-modified silica gel with a 5–7 *μ*m particle size, flow rate of 1.3 mL/min, mobile phase acetonitrile:water (80:20, *v*/*v*), and sample volume of 10 *μ*L. The sugars were detected by a refractive index detector at 30°C with column temperature adjusted to 300°C. Identification of sugars in honey was done by comparing their retention time with that of the standard sugars [23].

#### 2.2.5. Electrical Conductivity

The electrical conductivity of a solution of 20 g dry matter of honey in 100 mL distilled water was measured using an electrical conductivity cell (BANTE Instrument-520 conductive and temperature meter, China). Potassium chloride solution (0.1 M) was prepared by dissolving 0.745 g of potassium chloride dried at 130°C in 100 mL sterile distilled water. Forty milliliters of the potassium chloride solution was transferred to a beaker. The conductivity cell was connected to the conductivity meter, rinsed thoroughly with potassium chloride solution, and immersed in the solution together with a thermometer. The electrical conductance was read in mS after the temperature was equilibrated to 20°C [23].

The cell constant *K* was calculated using the following formula:

 $$\begin{array}{c} K=11.691\times \frac{1}{G} \end{array}$$

where *K* is the cell constant in cm^−1^, *G* is the electrical conductance in millisiemen, measured with the conductivity cell, and 11.691 is the sum of the mean value of the electrical conductivity of distilled water in mS.cm^−1^ and the electrical conductivity of a 0.1 M potassium chloride solution at 20°C.

#### 2.2.6. Determination of Hydroxymethyl Furfural (HMF)

HMF was determined using a 6800 UV–Vis spectrophotometer (Jenway, United Kingdom). Five grams of honey sample was weighed in a small beaker, mixed in a 25 mL distilled water, and transferred into a 50 mL volumetric flask [23]. Carrezz Solution I (0.5 mL) (15 g K_4_Fe (CN) _6_. 3H_2_O/100 mL distilled water) was added and mixed into 0.5 mL Carrezz Solution II (30 g) Zn(CH_3_.COO)_2._ 2H_2_O)/100 mL distilled water). The solution was mixed with the honey solution, and a drop of ethanol was added to the solution to suppress the foam. The solution was filtered through filter paper, and the filtrate (10 mL) was discarded. Filtrate (5 mL) was added into each of the two test tubes, and 5 mL distilled water was added to the first test tube (sample solution), while 5 mL sodium bisulfite solution (0.2%) of NaHSO_3_/100 mL distilled water) was added to the other test tube. The contents of both test tubes were mixed well by a vortex mixer and their absorbance was recorded spectrophotometrically at wavelengths of 284 and 336 nm. The result was expressed according to the International Honey Commission by subtracting the absorbance measured at 336 nm from that of 284 nm [23]. The HMF/100 g honey was calculated as

 $$\begin{array}{c} \frac{\text{HMF}}{100\text{ g honey }}=\left(\text{A}284-\text{A}336\right)\times 14.97\times \frac{5}{\text{g sample}} \end{array}$$

where *A*_284_ is the absorbance at 284, *A*_336_ is the absorbance at 336, 14.97 is the constant, 5 is the theoretical nominal sample weight, and *g* is the mass of honey sample.

### 2.3. Antioxidant Activities

#### 2.3.1. Total Phenolic Contents

To analyze and compare the total phenol content between honey samples, the Folin–Ciocalteu method was used [24]. A honey stock solution was prepared by mixing 5 g of honey sample in 50 mL of distilled water and filtered through Whatman No. 1 filter paper. From this stock solution, a 0.5 mL aliquot was mixed with 2.5 mL of 0.2 N Folin–Ciocalteu reagent and incubated for 5 min. Two milliliters of 75 g/L sodium carbonate solution was added to the solution. Finally, after the solution was incubated for 2 h at 25°C, the absorbance of the reaction mixture was measured at 765 nm using a UV spectrophotometer (PerkinElmer Lambda 950 UV/VIS/NIR). Gallic acid (0–200 mg/L) was used as a standard to produce a calibration curve, and finally, the total phenol content was expressed as milligrams of gallic acid equivalent (GE) in 100 g of honey from the mean value of triplicate data.

#### 2.3.2. Total Flavonoid Content (TFC)

The TFCs of honey samples from each source were determined [24]. The stock solution was prepared by diluting 5 g of honey sample in 50 mL of distilled water and filtered through Whatman No. 1 paper. Five milliliters of the honey stock solution was pipetted and mixed in 5 mL of a 2% aluminum chloride (AlCl_3_) solution. After incubation for 10 min, the absorbance of the reaction mixture was measured at 415 nm by using a spectrophotometer (Perkin Elmer Lambda 950 UV/VIS/NIR spectrophotometer). Quercetin (0–200 mg/L) was used as a standard chemical to produce a calibration curve, and finally, the TFC was reported as the mean value of triplicate assays and expressed as milligrams of Quercetin equivalent (QE) per 100 g of honey from the mean value of triplicate data.

#### 2.3.3. Antioxidant Content of Honey

Antioxidant compounds in honey samples were evaluated by measuring the ascorbic acid equivalent antioxidant capacity (AAEAC) following the standard method [25]. The DPPH (2,2-diphenyl-1-picrylhydrazyl) solution (20 mg L^−1)^ was prepared by dissolving 0.5 mg of DPPH in 25 mL of methanol. The honey solution was prepared by mixing 30 mg honey in 1 mL methanol, and 0.75 mL of methanolic honey solution was added to 1.5 mL of DPPH solution. The absorbance was measured at 517 nm after 15 min of incubation at room temperature. The blank was composed of 0.75 mL of a methanolic honey solution mixed with 1.5 mL of methanol. Ascorbic acid (0–200 mg L^−1)^ was used as a standard to produce a calibration curve. Finally, the measurements were replicated three times, and the mean value was expressed as milligrams of ascorbic acid equivalent antioxidant content per 100 g of honey from the mean value of triplicate data.

### 2.4. Data Analysis

Data (means ± SD) were calculated using SAS software (SAS Institute, 2003; 14). Significant differences in physicochemical and antioxidant parameters of the tested honey samples were obtained with one-way ANOVA, and means were separated with LSD tests. *p* < 0.05 was considered statistically significant.

## 3. Result and Discussion

### 3.1. Physicochemical Properties of Fresh and Market Honeys

#### 3.1.1. Moisture Content

The mean moisture content of fresh *A. mellifera* (AMFH) fresh honey directly collected from hives was in agreement with the values reported by Tigistu, Worku, and Mohammed [18] and Gebremedhin, Tadesse, and Kebede [26], but higher than the moisture content of fresh honey collected from modern (15.3 ± 1.03) as well as traditional hives (16.6 ± 1.14) as reported by Alemu, Seifu, and Bezabih [27]. AMMH had significantly higher mean moisture content (22 ± 3.0, *p* < 0.0001) compared to the fresh honey samples from hives (Table 1). Tigistu, Worku, and Mohammed [18] also reported significantly higher moisture content of honey samples from shops compared to those from farms in the southern part of Ethiopia. The high moisture content was also in agreement with the value reported by Kinoo, Mahomoodally, and Puchooa [28] from Mauritius. The mean moisture content of AMFH was in agreement with national standard [7] and international [17, 29] parameters recommended for *A. mellifera* honey. However, the moisture content of AMMH was found to be slightly above the national and international standards.

The differences in the moisture contents of the fresh honeys and market honeys could be attributed to differences in the harvesting procedures, storage conditions, seasons of collection, and level of maturity [18, 30]. According to Ordó, González, and Escobedo [31], honey harvested during the rainy seasons contained more moisture than those harvested during the dry seasons.

The moisture content of MBH in the present study was in a range reported by Damto, Kebede, and Gemeda [32] from Ethiopia for the same stingless bee species and de Souza et al. [33] from Brazil for a different stingless bee species, but slightly higher than the moisture content reported by Alvarez-Suarez et al. [34], Andualem [35] for honey from *Trigona* species, and dos Santos et al. [36] and Zawawi et al. [37] for *Tetragonula* species.

The moisture content of MBH from the market sources in the current study was significantly higher (*p* < 0.0001) than the value for *A. mellifera* honey from the same sources (Table 1). Similarly, higher moisture contents of SBH honey compared to *A. mellifera* honey were reported by Kek et al. [38] and de Sousa et al. [33]. Factors such as bee species, floral source, honey harvesting time, the degree of maturity achieved in the hive, and climatic factors affect the moisture content of honey [39].

#### 3.1.2. Free Acidity, pH, and HMF

Free acidity did not significantly differ between fresh *A. mellifera* honey and those from market sources (Table 1). The values obtained in the current study were in a range reported from Tigray [26] and Amhara [27] but higher than the values reported for honey from the same species from Nigeria [40] and Polish markets [23]. The mean free acidity of both market and fresh honey samples in the current study was in agreement with the national [7] and international [6, 29] quality standards of *A. mellifera* honey. None of the honey samples from *A. mellifera* exceeded the acceptable limit.

The free acidity of the stingless bee honey from market sources was significantly higher than *A. mellifera* honey from the same source (*p* < 0.0001) (Table 1). The highest mean free acidity value of 113 ± 5 meq/kg^−1^ was recorded for MBH. The free acidity value in the current study was higher than the values previously reported [32, 34, 40] but comparable with the Malaysian stingless bee (*Heterotrigon itama*) [38], Australian stingless bee (*Tetragonula carbonaria*) [41], and *Melipona quadrifasciata* and *Tetragonisca angustula* from Brazil [42], respectively. The free acidity of MBH was 4.5 times higher than *A. mellifera* honeys from the market sources, which is in agreement with the values reported by other studies [38, 43]. The free acidity of honey from the stingless bee (MBH) is lower than the values reported for *H. itama* from Malaysia [42].

Free acidity indicates one of the quality parameters of honey samples, and it reveals whether the honey is fermented or not [43] and corresponds to the presence or absence of organic acids in the product. The differences in the free acidity of honey samples produced by different bee species might be due to the fermentation of sugar into organic acids [43].

The MBH samples had pH values of 3.0 ± 0.0 which is comparable with the values reported for *M. beccarii* by Damto, Kebede, and Gemeda [32] from Ethiopia and Alvarez-Suarez et al. [34] for stingles bees from Cuba (*Meliponula species*), but higher than the value reported by Andualem [35] from a *Trigona* species, Souza et al. [42] from *H. itama*, and dos Santos et al. [36] from *Tetragonula* species.

The mean pH values of honey (*A. mellifera*) observed from the present investigation were within the pH range of international standard (3.2–4.5) and comparable with Malaysian honeys (pH = 3.78 ± 0.21) [44], but lower than the pH value from the Istanbul market (pH = 4.32) [45]. The differences in the pH values of the samples might be due to the differences in the source of honey such as entomological, botanical, and processing methods [34].

The highest concentration of HMF (15.5 ± 8 mg/kg^−1^) was recorded for AMMH. The amount of HMF of AMMH significantly (*p* < 0.01) differed from AMFH and MBH (Table 1). The amount of HMF and enzymatic activity in honey is one of the important indicators of honey's quality (freshness), indicating whether the honey is aged or overheated [46]. Variability in the HMF values of the stingless bee (*M. beccarii*) honey was reported by Damto, Kebede, and Gemeda [32], with most values higher than the HMF value we reported in this research. A much higher value of HMF was reported for honey from *H. itama*, a stingless bee from Malaysia, by Ramlan et al. [47]. But none of the investigated samples exceeded the allowed limit of national [7] and international [6, 29] quality standards (Table 1). But the need for specific quality standards for stingless bee honey including the use of the newly discovered trehalulose sugar was recommended [36, 37, 47].

From the present study, honey samples from the market had low free acidity, high pH, and high HMF compared to fresh honey directly harvested from modern hives and underground nests of stingless bees. The differences observed might be associated with beekeepers or honey retailers processing methods such as exposure to heating or sunlight, which deactivate glucose oxidase activity, limiting the production of acids. The differences in the amount of HMF were also reported by Chua et al. [24]. According to these authors, the HMF content of honey increases due to overheating, aging, and poor storage conditions. The EU's regulation suggests that HMF content should not be more than 40 mg/kg [48].

#### 3.1.3. Ash Content and Electrical Conductivity

The average ash contents of different honey samples are indicated in Table 1. Significant variation was not observed in the ash contents of fresh and market honeys. The mean ash content (g/100 g) of AMMH, AMFH, and MBH were 0.33 ± 0.15, 0.40 ± 0.67, and 0.26 ± 0.10, respectively. The ash contents of AMFH and AMMH samples fell within the value recommended by the International Honey Commission [6, 29], which is not more than 0.6% for *A. mellifera*, and the National Standard value of ≤ 0.5. Similarly, the ash content of MBH was found within the concentration proposed by Vit, Medina, and Enríquez [43], who suggested that the ash content of honey from stingless bees (*M. beechei*) should not be more than 0.5% (Table 1). The ash content MBH of this study was lower than the result reported by Alvarez-Suarez et al. [34] from stingless bees (*M. beechei*). The differences in the ash content might be due to floral origin and soil features [49]. The blossom honeys have a lower ash content than the honeydew [23].

The electrical conductivity (millisiemens per centimeter) of AMFH significantly (*p* < 0.01) differed from the EC values of AMMH and MBH (Table 1). Electrical conductivity is determined by the ability of ions present in a sample to conduct electrons, and it has been found to assist in the determination of the botanical origin of honey samples. The electrical conductivity of honey from the three sources fell within the value recommended by the International Honey Commission [6] and Ethiopian standards [7], with the maximum EC value of 0.8% for honey from *A. mellifera* (Table 1). The electrical conductivity of MBH in this study was less than the values reported by Damto, Kebede, and Gemeda [32] for *M. beccarii* honey, Alvarez-Suarez et al. [34] for *M. beecheii* honey, and the value reported for honey from *Trigona* [35] and Tetragonula species [36]. However, the value is comparable with a report by Ramlan et al. [47] for honey from *H. itama*.

Generally, as the value of electrical conductivity increased, the ash content of the honey samples also increased for each source of honey treatments, which is in agreement with the result reported by Kek et al. [38]. Similar relationships between electrical conductivity and ash content of honey samples were also reported by Acquarone, Buera, and Elizalde [50]. According to Biluca et al. [51], variations in the electrical conductivity of honey samples were linked to differences in the geographical, botanical, and entomological origins of the honey samples.

### 3.2. Reducing Sugar (Glucose, Fructose, and Maltose) and Nonreducing Sugar

The average concentration of reducing sugars (sum of glucose, fructose, and maltose) of honey samples analyzed was 70 ± 3.0% for AMMH, 75 ± 2.5% for AMFH, and 41 ± 5.0% for MBH. A significant difference (*p* < 0.05) was observed between samples (Table 2). The highest reducing sugar recorded was for fructose, but the difference between fresh and market honey was not statistically significant. The overall concentration of reducing sugars in fresh honey was significantly higher than that of market honey. Similarly, a higher concentration of fructose was also reported for honey samples from different sources [52, 53]. The glucose content of both fresh and market honey in our study was lower than that of fructose. According to Escuredo et al. [53], the amount of glucose was 3%–6% lower than the fructose content. The concentration of reducing sugar from AMFH in this study was higher than reports from Tigray, Ethiopia [26], and Amhara, Ethiopia, from *A. mellifera* [27]. Similarly, a very close concentration of reducing sugar to the concentration in the current study was also reported for honey samples from Nigeria [40], but the concentration of reducing sugar from AMFH and AMMH of this study was higher than the concentration of reducing sugar reported for Malaysian honey [44] and Bangladesh [25]. The concentration of total reducing sugar for honey from the stingless bee in the current study was less than the value reported by Damto, Kebede, and Gemeda [32]. The principal carbohydrate constituents of honey are fructose and glucose, which represent 85%–95% of total sugars that are readily absorbed in the gastrointestinal tract [54, 55]. Generally, from this study, fructose and glucose sugars are the dominant sugars found in all sources of honey samples, while fructose was more abundant than glucose. This is also supported by research results from Nigeria [54], Turkey [56], and Poland [57] for *A. mellifera* honey samples. All the honey samples from *A. mellifera* were within the permitable value of the national [7] and international quality requirements [6, 29].

The MBH collected from the market had significantly lower sugar content compared to the *A. mellifera* honeys collected from market (Table 2). The reducing sugar produced from MBH of the present study was much less and did not fit the standard proposed by some investigators—more than 50% by Vit, Medina, and Enríquez [43], 63% by Fonte et al. [58], and 76% by Nweze et al. [40] for honey from the same species of stingless bee. The presence of enzymes in bees and nectar, as well as the presence of sugars in the nectar of plants, are the main factors for the sugar in any honey [59]. A similar pattern in the reducing and nonreducing sugar content was also reported by Damto, Kebede, and Gemeda [32] for similar species of stingless bees. Chuttong et al. [60] reported reducing sugar content to less than 60%, which is in agreement with the result of our study. Recent studies reported the predominant presence of reducing sugar, trehalulose, in the honey samples collected from Malaysia and Australia [36, 37, 47]. In the stingless honey, fructose and glucose were reported to be the predominant reducing sugars, respectively, next to trehalulose [36, 47]. Differences in the trehalulose content between different stingless bee species were also reported [37]. But differences were not found in the trehalulose content across different geographical locations [47]. Deviations in the moisture content, free acidity, total glucose, and fructose levels were also reported [37]. The lower sugar content of honey from stingless bees is mainly due to botanical origin [61].

The water content of the MBH from the market sources was significantly higher than that of the corresponding AMMH, which could give space for the hydrolysis of fermentable sugars by spoilage microorganisms. The amount of rainfall also affects the concentration of sugars in honey, with more sugars available in the plant in the dry season than in the rainy season [62].

All of the honey samples fit the values of national [7] and international [8, 29] quality requirements with a maximum of 10% and 5% sucrose content from *A. mellifera*, respectively. Sucrose concentrations from honey samples in the current study were lower than the values reported from Malaysia [44], Bangladesh [25], and Nigeria [40]. A study by dos Santos et al. [36] and Ramlan et al. [47] reported the complete absence of sucrose in honey samples tested. The sucrose content in mature honeys could be low due to invertase enzyme activity that degrades sucrose [58]. The conversion of sucrose to trehalulose was used to justify the complete absence of sucrose from stingless bee honey [36]. Adulteration was not detected in the honey samples evaluated, as can be understood from the low level of sucrose in the current study, as adulteration with table sugar could increase the sucrose level above the normal values.

### 3.3. Antioxidant Activity of Fresh and Commercial Honey From *A. mellifera*

#### 3.3.1. Total Phenolic Content (TPC)

The honey samples from commercial sources did not significantly differ in their TPC compared to fresh honey collected from hives (Table 3).

Numerically high TPC was recorded for the MBH sample with a mean value of 273 ± 9 mgGAE/100 g^−1^, followed by AMMH (149 ± 6 mgGAE/100 g^−1^) and AMFH (138 ± 7 mgGAE/100 g^−1^). Compared to the TPC values from other countries, the mean values of TPC for *A. mellifera* honey in this study were higher than those of Malaysian samples (186.70 ± 0.84 mgGAEkg^−1^) from Acacia forage and (226.29 ± 1.18 mgGAEkg^−1^) from pineapple forage [44], from Burkina Faso [8] and Cuba [34]. However, the TPC of honey from *A. mellifera* in this study was less than Sudanese honey (201.08 ± 2.49 mgGAE/100 g^−1^ honey) [63] and market honey from Egypt (550–1412 mgGAE/100g^−1^) [64]. Chemical composition and TPC are generally dependent on bee species, bee forage, and the geographical origin of honey [65].

From the present study, MBH contained more total phenol compared to honey samples from *Apis* species. This is in agreement with the investigation by Alvarez-Suarez et al. [34], who reported that both *A. mellifera* and *M. beecheii* are important sources of bioactive compounds, but honey from *M. beecheii* had the highest total phenol contents. The authors also concluded that the biological properties and composition of honey samples depended on the bee species. Furthermore, Kek et al. [38] reported higher TPC from honey samples of the Malaysian stingless bees (*H. itama*) compared to the TPC of honey from the *Apis* spp. Similarly, the TPC of honey from Plebeia stingless bees was slightly higher than those from *A. mellifera* [66].

Higher TPC was recorded for MBH from this study compared to reports of TPC from polyfloral honey from the stingless bee, *M. beecheii*, from Cuba [34] and TPC from the stingless bee, *Hypotrigona* spp., from the Nigerian Nsukka sample [40], and for honey from *Tetragonula* species from Australia [36]. The total phenolic compounds vary between honey samples and depend on the bee species and floral and geographical origin of the honey [65].

#### 3.3.2. TFC

The highest TFC was recorded for MBH, followed by AMMH (Table 3). The TFC from this study was higher than the report from the Turkish honey from *A. mellifera* [56] and commercial Portuguese honeys [67]. The TFC in the current study was similar to the TFC contents of honey from Portuguese healthcare samples [67]. Phenolic acid, flavonoid, and tannins are the common classes found under polyphenol [68] with wide structural differences.

MBH had the highest TFC contents compared to honey from the other two sources in the current study as well as honey from the Cuban stingless bee (*M. beecheii*) [34]. *M. beccarii c*ommonly visits herbaceous plants such as *G. scabra* and *P. lanceolate*. Thus, the plant species, in addition to the bee species, might be the source of high TFC detected from the honey samples. It was investigated that honey samples of monofloral (*Guizotia abyssinica*) origin had high antioxidant properties compared to Bangladesh honeys from other sources [25]. The differences between the results of the current study and other studies might be due to differences in the source of honey and methodologies employed in addition to the honeybee types and geographical locations.

High TFC and TPC were obtained from AMMH compared to AMFH. The aggregation of compounds that are released from different plants such as herbaceous, shrubs, and tree types, might cause the *A. mellifera* market honey (multifloral honey) to have more TFC and TPC than honey collected directly from hives, which was monofloral honey (*C. arabica*). A similar study reported that multifloral honeys have higher antioxidant properties based on their high levels of phenolics, flavonoids, the ascorbic acid equivalent of antioxidant content (AEAO), and DPPH compared to monofloral honeys [25].

#### 3.3.3. Antioxidant Content

Statistically, no variation (*p* > 0.05) was observed between all sources of honey samples with regard to antioxidant content. However, the highest concentration of antioxidants (104 ± 6 mgAAE/100 g^−1^) was recorded for MBH samples while the lowest (42 ± 6 mgAAE/100 g^−1^) for AMFH (Table 3). The mean values of antioxidants from AMFH were within the range reported from the Burkina Faso honey sample from *A. mellifera* [8], while higher than Bangladesh honeys from *A. mellifera* [25] and Indian honey [69].

The result of the antioxidant content from the present study is in agreement with Kek et al. [38], who reported that honey from Malaysian stingless bees (*H. itama*) had higher antioxidant properties compared to honey from *Apis* spp. Likewise, Meda et al. [8] reported that honey with the highest ascorbic acid equivalent (AEAC) had the highest antioxidant activity. Honey from stingless bees (MBH) in the current study exhibited higher values of TPC, TFC, and AOC and showed better antioxidant properties compared to honey produced by *A. mellifera*. Similarly, Kelulut honey produced by the stingless bees from the *Trigona* stingless showed significantly higher values in moisture content, water activity, and free acidity with better antioxidant properties (high TPC and TAEAC) compared to honey produced by *Apis* spp. [38].

## 4. Conclusion

*A. mellifera* honey samples harvested directly from the hive and purchased from the local market of the study area showed good quality in regard to their physicochemical properties based on recommendations by international (European Union Commission and Codex Alimentarius) and national (Ethiopian Standard Agency) standards. The antioxidant content of fresh and market honey did not differ significantly, though the antioxidant content of stingless bee honey from the markets was higher than *A. mellifera* honey from the same source. The *A. mellifera* honey samples collected directly from hives had better quality compared to the samples purchased from marketplaces. Training for the producers and stringent market assessment methods and regulations should be put in place in order to prevent quality loss.

## Figures and Tables

**Table 1:** Physicochemical composition of honey from different sources.

**Parameter**	**Sources of honey samples (mean ± SD)**			**X**	**LSD**	**p** ** value**	**Standard**		
	**AMMH**	**AMFH**	**MBH**				**Codex Alimentarius (2001) *A. mellifera***	**EU Council (2002) *A. mellifera***	**Ethiopia *A. mellifera***
MC (%)	22^b^ ± 3.0	18.64^c^ ± 1.0	33.5^a^ ± 3.0	24.63	2.24	< 0.0001	≤ 20	≤ 20	≤ 20
FA (meq/kg)	25.3^b^ ± 2.5	33^b^ ± 1.6	113^a^ ± 5.0	57.87	12.76	< 0.0001	≤ 50	≤ 50	≤ 50
pH	3.8^a^ ± 0.0	3.6^b^ ± 0.1	3.0^c^ ± 0.0	3.48	0.10	< 0.0001	—	—	—
HMF (mg/kg)	15.5^a^ ± 8.0	5.6^b^ ± 3.0	4.4^b^ ± 2.0	8.51	5.36	0.0003	≤ 40	≤ 40	≤ 40
ASH (g/100 g)	0.3^a^ ± 0.0	0.4^a^ ± 1.0	0.26^a^ ± 0.0	0.33	0.37	0.7498	≤ 0.6	≤ 0.6	≤ 0.5
EC (mS/cm)	0.26^b^ ± 0.1	0.38^a^ ± 0.0	0.25^b^ ± 0.0	0.29	0.06	0.0003	≤ 0.8	—	≤ 0.8

*Note:* Means with different superscript letters (a, b, c) within the rows are statistically different at *p* ≤ 0.05. AMFH = *A. mellifera* fresh honey directly collected from hives, AMMH = *A. mellifera* honey collected from the local markets, ASH (g/100 g) = ash content of honey in g/100 g, EC (mS/cm) = electrical conductivity in millisiemens per centimeter, FA (meq/kg) = free acidity in mill equivalents per kilogram of honey, HMF (mg/kg) = hydroxymethyl furfural in milligrams per kilogram of honey, LSD = least significant difference at alpha = 0.05, MC (%) = moisture content of honey in percent, MBH = *M. beccarii* honey collected from markets, X = overall mean.

Abbreviation: SD = standard deviation.

**Table 2:** Sugar contents of honey samples from different sources.

**Sugar**	**Sources of honey samples (mean ± SD)**			**X**	**LSD**	**p** ** value**	**Standard**		
	**AMMH**	**AMFH**	**MBH**				**Codex Alimentarius *A. mellifera***	**EU Council *A. mellifera***	**Ethiopia *A. mellifera***
Fructose (%)	38.8^a^ ± 2.6	38.6^a^ ± 0.5	19.6^b^ ± 2.4	32.35	1.93	< 0.0001	—	—	—
Glucose (%)	30.8^b^ ± 3.37	33.5^a^ ± 1.54	18.2^c^ ± 1.62	27.54	2.14	< 0.0001	—	—	—
Maltose (%)	0.7^b^ ± 0.1	2.6^a^ ± 0.7	3.0^a^ ± 1.0	2.11	0.64	< 0.0001	—	—	—
RS (%)	70^b^ ±3.0	75^a^ ± 2.5	41^**c**^ ± 5.0	61.82	2.90	< 0.0001	≥ 60	≥ 60	≥ 60
Sucrose (%)	1.03^a^ ± 0.50	0.26^ab^ ± 0.32	0.18^b^ ± 0.13	0.49	0.81	0.08	≤ 5	≤ 5	≤ 5

*Note:* Means with different superscript letters (a, b, c) within the rows are statistically different at *p* ≤ 0.05. AMFH = *A. mellifera* fresh honey directly collected from hives, AMMH = *A. mellifera* honey collected from the local markets, LSD = least significant difference at alpha = 0.05, MBH = *M. beccarii* honey collected from markets, RS (%) = percent of reducing sugar in honey, X = overall mean.

Abbreviations: Fru = fructose, Glu = glucose, Mal = maltose, SD = standard deviation.

**Table 3:** Antioxidant properties of honey.

**Parameters**	**Sources of honey sample (mean ± SD)**			**X**	**LSD**	**p** ** value**
	**AMMH**	**AMFH**	**MBH**			
TPC (mgGAE/100 g of honey)	149^a^ ± 6	138^a^ ± 7.0	273^a^ ± 9.0	183.3	165.9	0.1507
TFC (mgQE/100 g of honey)	31^a^ ± 4	22^a^ ± 10.0	41^a^ ± 21.0	31.3	32.3	0.4145
AOC (mgAAE/100 g of honey)	93^ab^ ± 4	42^b^ ± 6.0	104^a^ ± 6.0	83.2	62.1	0.5538

*Note:* Means with the same superscript letter (a, b) within the row are not statistically significant (*p* > 0.05). AMFH, *A. mellifera* fresh honey directly collected from hives; AMMH, *A. mellifera* honey collected from the local markets; AOC (mgAAE/100 g of honey), antioxidant content in milligram of ascorbic acid equivalent in 100 g of honey sample; LSD, least significant difference at alpha = 0.05; MBH, *M. beccarii* honey collected from markets; TFC (mgQE/100 g of honey), total flavonoid content in milligrams of quercetin equivalent per 100 g of honey sample; TPC (mgGAE/100 g of honey), total phenolic content in milligrams of gallic acid equivalent per 100 g of honey sample; X, overall mean.

Abbreviation: SD, standard deviation.

## Data Availability

All the necessary data have been included in the manuscript.
